# Synthesis of ^11^C‐labeled ubiquinone and ubiquinol via Pd^0^‐mediated rapid C‐[^11^C]methylation using [^11^C]methyl iodide and 39‐demethyl‐39‐(pinacolboryl)ubiquinone

**DOI:** 10.1002/jlcr.3700

**Published:** 2019-01-08

**Authors:** Miki Goto, Akira Nishiyama, Takao Yamaguchi, Kyosuke Watanabe, Kenji Fujii, Yasuyoshi Watanabe, Hisashi Doi

**Affiliations:** ^1^ Laboratory for Labeling Chemistry RIKEN Center for Biosystems Dynamics Research Kobe Japan; ^2^ Pharma & Supplemental Nutrition Solutions Vehicle Kaneka Corporation Takasago Japan; ^3^ Laboratory for Pathophysiological and Health Science RIKEN Center for Biosystems Dynamics Research Kobe Japan; ^4^ Department of Physiology, Graduate School of Medicine Osaka City University Osaka Japan; ^5^ Pharma & Supplemental Nutrition Solutions Vehicle Kaneka Corporation Tokyo Japan

## Abstract

To enable positron emission tomography (PET) imaging of the in vivo kinetics of ubiquinone and ubiquinol, which is referred to as coenzyme Q_10_, their ^11^C‐radiolabeled counterparts were synthesized herein. ^11^C‐Labeled ubiquinone [^11^C]‐**1** was realized by Pd‐mediated rapid *C*‐[^11^C]methylation of [^11^C]CH_3_I with 39‐demethyl‐39‐(pinacolboryl)ubiquinone, prepared by Ru‐catalyzed olefin metathesis of unradiolabeled ubiquinone with 2‐(pinacolboryl)propene. Subsequent reduction of [^11^C]‐**1** using Na_2_S_2_O_4_ yielded ^11^C‐labeled ubiquinol [^11^C]‐**2**. The synthesis time and [^11^C]CH_3_I‐based radiochemical yield of [^11^C]‐**1** were within 36 minutes and up to 53%, while those of [^11^C]‐**2** were within 38 minutes and up to 39%, respectively. After radiopharmaceutical formulation, the qualities of [^11^C]‐**1** and [^11^C]‐**2** were confirmed to be applicable for animal PET studies. The analytical values of [^11^C]‐**1** and [^11^C]‐**2** are as follows: radioactivity of up to 3.5 and 1.4 GBq, molar activity of 21 to 78 and 48 to 76 GBq/μmol, radiochemical purity of greater than 99% and greater than 95%, and chemical purity of greater than 99% and 77%, respectively. The concept behind this radiolabeling procedure is that unradiolabeled natural ubiquinone can be converted to ^11^C‐radiolabeled ubiquinone and ubiquinol via a pinacolborane‐substituted ubiquinone derivative. Each PET probe was used for molecular imaging using rats to investigate the in vivo kinetics and biodistribution of the coenzyme Q_10_.

## 1 INTRODUCTION

**Scheme 1:**
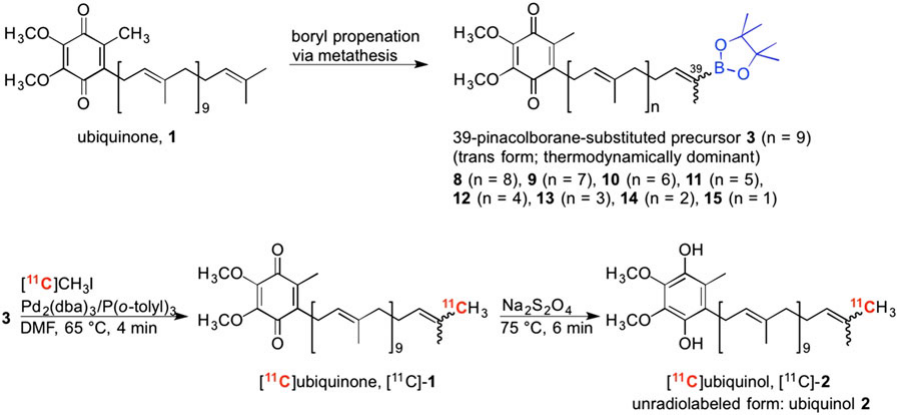
Synthesis of [^11^C]ubiquinone and [^11^C]ubiquinol from ubiquinone

Ubiquinone **1** and ubiquinol **2**, referred^1^, ^2^, ^3^, ^4^, ^5^, ^6^ to as coenzyme Q_10_, exhibit a chemically intriguing framework of long polyprenylated side chains (10 units) functionalized onto benzoquinone or benzoquinol units. According to their redox states, ubiquinone and ubiquinol are classified into oxidized and reduced forms, respectively. Indeed, these compounds, which are described as vitamin‐like substances, play crucial roles in controlling the antioxidant activity to protect cells from free radicals and act as essential electron carriers in mitochondria, which converts the energy in carbohydrates and fatty acids into ATP.^1^, ^2^, ^3^, ^4^, ^5^, ^6^ A healthy human body maintains regular biosynthetic functions to produce enough amounts of ubiquinone and ubiquinol, but their production tends to decrease with chronological age.^4^, ^5^, ^6^ Therefore, dietary or supplemental intake of ubiquinone and ubiquinol is recommended with advancing age. However, there is little information on their in vivo pharmacokinetics, and the difference between ubiquinone and ubiquinol in living systems is not well known. Therefore, in this investigation, we focused on using positron emission tomography (PET), which is capable of recording dynamic images of accumulation and distribution of ubiquinone and ubiquinol in tissues or the whole body. Here, we report the synthesis of ^11^C‐labeled ubiquinone [^11^C]‐**1** and ubiquinol [^11^C]‐**2** via Pd‐mediated rapid *C*‐[^11^C]methylation^7^, ^8^, ^9^ of [^11^C]CH_3_I^10^, ^11^, ^12^, ^13^, ^14^, ^15^, ^16^, ^17^ with a corresponding pinacolboryl precursor **3** and continuous reduction with Na_2_S_2_O_4_ (Scheme 1). Indeed, the synthetic method represents that unradiolabeled ubiquinone undergoes chemical reactions of metathesis^18^, ^19^ and ^11^C‐radiolabeling to eventually afford terminally [^11^C]CH_3_‐incorporated ubiquinone and ubiquinol.

## 2 RESULTS AND DISCUSSION

**Scheme 2:**
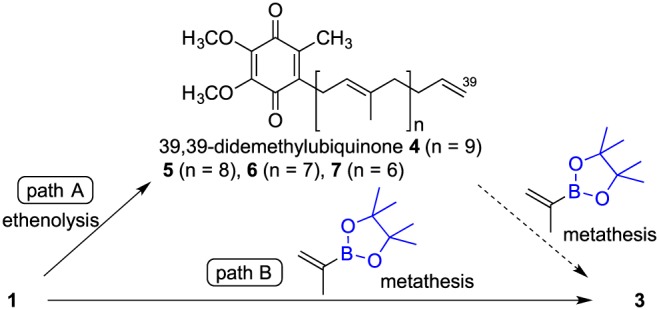
Preparation of pinacolborane **3** via olefin metathesis of ubiquinone

The radiochemistry group of Tohoku University already reported the synthesis of ^11^C‐labeled ubiquinone by 5‐*O*‐[^11^C]methylation of the quinone structure using 5‐*O*‐demethyl ubiquinone as a substrate.^20^, ^21^ On the other hand, we concentrated on the synthesis by *C*‐[^11^C]methylation at the terminal position of the polyprenylated side chains of ubiquinone. We considered that the use of 5‐*O*‐[^11^C]CH_3_‐incorporated or 39‐*C*‐[^11^C]CH_3_‐incorporated ubiquinone could give us the opportunity to investigate not only the in vivo kinetics but also the metabolic pathway of ubiquinone as exemplified by their oxidation via cytochrome P450 and 5‐*O*‐demethylation via demethylase.^22^ Therefore, with the objective of carrying out another efficient ^11^C‐labeling of ubiquinone and ubiquinol, while taking into consideration the short half‐life of ^11^C (20.4 min), we focused on 39‐demethyl‐39‐(pinacolboryl)ubiquinone **3**, as an essential precursor for Pd‐mediated rapid *C*‐[^11^C]methylation, which was initially planned via a two‐step metathesis consisting of ethylenation and borylpropenation (Scheme 2, path A). A study of previously reported metathesis methods for introducing vinylborane^23^ indicated that the less hindered terminal ethylene of 39,39‐didemethyl ubiquinone **4** is a requisite moiety, owing to the reactivity required for olefin metathesis with 2‐(pinacolboryl)propene.

**Figure 1:**
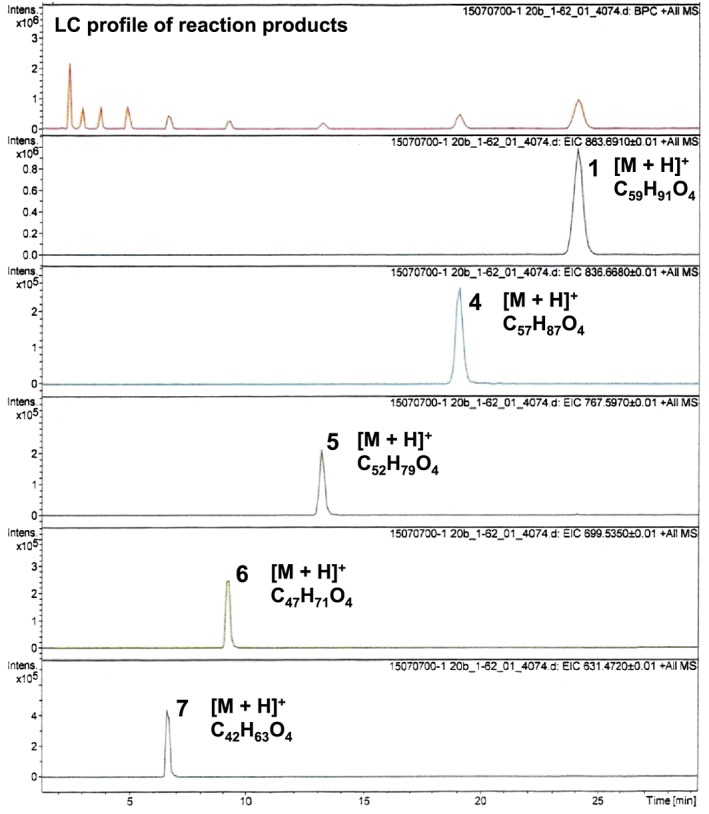
Liquid chromatography/time‐of‐flight mass spectrometry (LC/TOF‐MS) analysis of products of Ru‐catalyzed ethenolysis of **1** (first step of path A). LC conditions—column: YMC‐Pack Pro C18 150 × 4.6 mm I.D., eluent: methanol/hexane = 9:1, flow rate: 1.0 mL/min, UV: 290 nm, and column temperature: 30°C

However, we encountered difficulties in synthesizing the terminal ethylene compound **4** by Ru‐catalyzed ethenolysis of **1** as the first step. As shown in Figure 1, the metathesis reaction of **1** (430 mg, 500 μmol) with ethylene (3 bar) in an autoclave apparatus in toluene (10 mL) at 100°C for 3 hours using a second‐generation Hoveyda‐Grubbs catalyst^24^ (0.8 mg, 1.3 μmol, approximately 0.3 mol%) resulted in nonselective ethenolysis compounds,^25^ including not only the desired compound **4** but also polyprenyl side‐chain‐shortened by‐products **5**, **6**, and **7**. The yield of **4** was up to 17%. The reaction conditions were further investigated by modulating the solvent (acetic acid, heptane, and methyl *tert*‐butyl ether), temperature (50°C‐120°C), time (0.5‐21 h), ethylene gas pressure (1‐3 bar), and catalyst content (0.1‐1.0 mol%), but we could not overcome the shortcomings related to low selectivity and reactivity. As it was difficult to obtain terminal ethylene **4** at a high yield, we adopted path B, which is a one‐step metathesis of **1** and 2‐(pinacolboryl)propene.

For the metathesis reaction in path B, three popular catalysts, the first‐generation Piers catalyst,^26^ second‐generation Hoveyda‐Grubbs catalyst,^24^ and the Stewart‐Grubbs catalyst,^27^ were used.

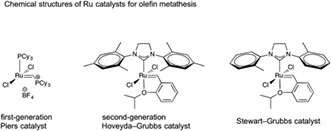

**Figure 2:**
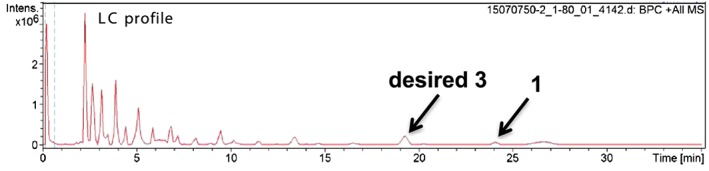
Liquid chromatography/time‐of‐flight mass spectrometry (LC/TOF‐MS) analysis of reaction products of metathesis between **1** and 2‐(pinacolbory)propene (path B)

Interestingly, usage of the second‐generation Hoveyda‐Grubbs catalyst resulted in a high reactivity and reaction at 70°C in toluene yielded the desired pinacolboryl compound **3** (up to 16% yield, Table 1, entry 3). However, as shown in the liquid chromatography/time‐of‐flight mass spectrometry (LC/TOF‐MS) analysis of the products (Figure 2), the molecular weights of each of the peaks indicated that a considerable amount of polyprenyl side‐chain‐shortened by‐products **8** to **15** (prenyl unit number: n = 1‐8) was produced and the total yield of the by‐products was 54%. The reaction conditions were further investigated in terms of the catalyst concentration and reaction time (Table 1, entries 1, 2, 4, and 5), but the yields of **3** were not very different from the result shown in entry 3. Meanwhile, reactions using the first‐generation Piers catalyst or the Stewart‐Grubbs catalyst resulted in **3** at trace amounts (less than 5%). Even though path B using the second‐generation Hoveyda‐Grubbs catalyst resulted in a low yield of 16%, we concluded that this one‐step procedure was useful for preparing **3** at a yield of several hundred milligrams.

**Table 1:** Olefin metathesis of 1 and 2‐(pinacolboryl)propene using the second‐generation Hoveyda‐Grubbs catalyst

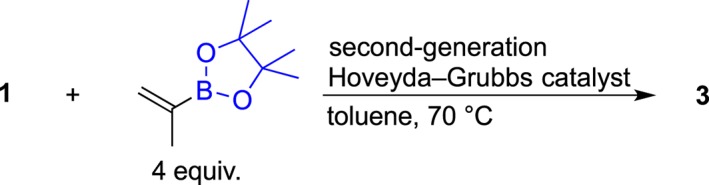					
Entry	Catalyst, mol%	Reaction Time, h	Recovery of **1**, %	Yield of Desired **3**, %	Yield of Other Products, %
1	1.0	39	10	10	15
2	2.0	2	14	14	20
3	3.0	2	16	16	56
4	5.0	3	11	11	26
5	5.0	3	15	15	54

The reaction of **1** (1.0 g, 1.2 mmol) with 2‐(pinacolboryl)propene (0.8 g, 4.8 mmol) in toluene (12 mL) was carried out at a specified reaction temperature and concentration (mol%) of the Hoveyda‐Grubbs catalyst, as shown in each entry of Table 1.

**Figure 3:**
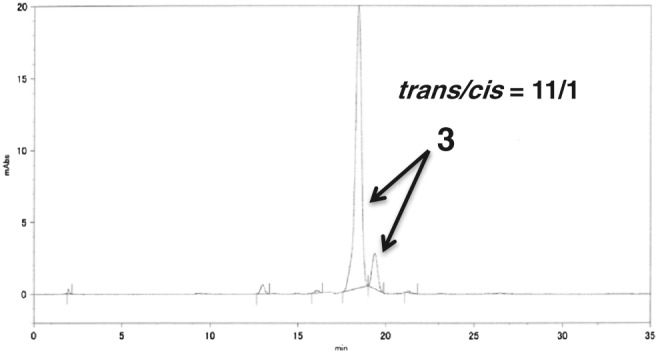
Purity analysis of the final product, **3**

To purify **3**, a metal‐ion scavenger was used to remove Ru metal from the reaction mixture. Later, the residue was purified by silica‐gel column chromatography to afford crude **3** with 28% purity. Subsequently, size‐exclusion chromatography was conducted to obtain **3** with 89% purity. In order to further increase the purity level, one more iteration of silica‐gel column chromatography was carried out to obtain greater than 97% pure **3** (Figure 3). High‐performance liquid chromatography (HPLC) analysis showed that the purified **3** was an 11:1 *trans*‐*cis* mixture with respect to terminal 2‐(pinacolboryl)propene (Figure 3).

**Figure 4:**
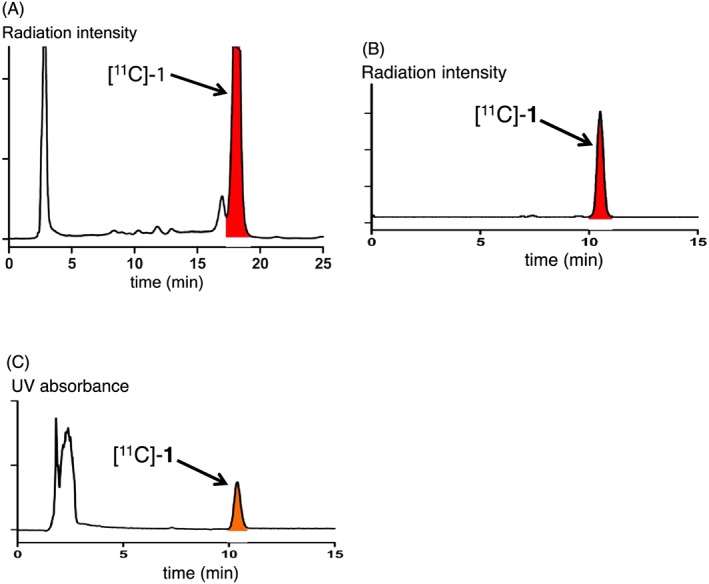
HPLC profiles of the reaction mixture and purity analysis during the synthesis of ^11^C‐labeled ubiquinone [^11^C]‐**1**. A, Semipreparative radio‐HPLC chromatogram; B, purity analysis of the injectable solution by radio‐HPLC; and C, purity analysis of the injectable solution by UV‐HPLC; the large peak at 1.5 to 3.5 min is mainly attributed to the use of propylene glycol and Tween 80 as additives

Using precursor **3**, the synthesis of ^11^C‐labeled ubiquinone [^11^C]‐**1** was carried out by Pd‐mediated^7^, ^8^, ^9^ rapid *C*‐[^11^C]methylation (Scheme 1). According to the procedure described in a previous report,^7^ we set up the ^11^C‐labeling conditions of [^11^C]CH_3_I (radioactivity: 20‐40 GBq), which included **3** (2.6 mg, 2.7 μmol), Pd_2_(dba)_3_ (1.4 mg, 1.5 μmol), and P(*o*‐tolyl)_3_ (1.9 mg, 6.2 μmol) in dimethylformamide (DMF, 450 μL). The reaction was carried out at 65°C for 4 minutes. As shown in Figure 4A, which illustrates a semipreparative HPLC chromatogram of the reaction mixture, the desired compound [^11^C]‐**1** was clearly indicated by a major peak. After radiopharmaceutical formulation using a solution of saline, propylene glycol, and Tween 80 (volume ratio: 100/10/0.5), the radioactivity and molar activity of [^11^C]‐**1** at the end of the synthesis were confirmed to be 1.5 GBq in normal cases (obtained radioactivity range: 0.4‐3.5 GBq) and 21 to 78 GBq/μmol, respectively. The total synthesis time was less than 36 minutes, and the radiochemical yield based on [^11^C]CH_3_I was up to 53%. The radiochemical purity and chemical purity were found to be greater than 99% (Figure 4B and 4C). The pH value was approximately 7. These qualities fully met the criteria for animal PET studies.

**Figure 5:**
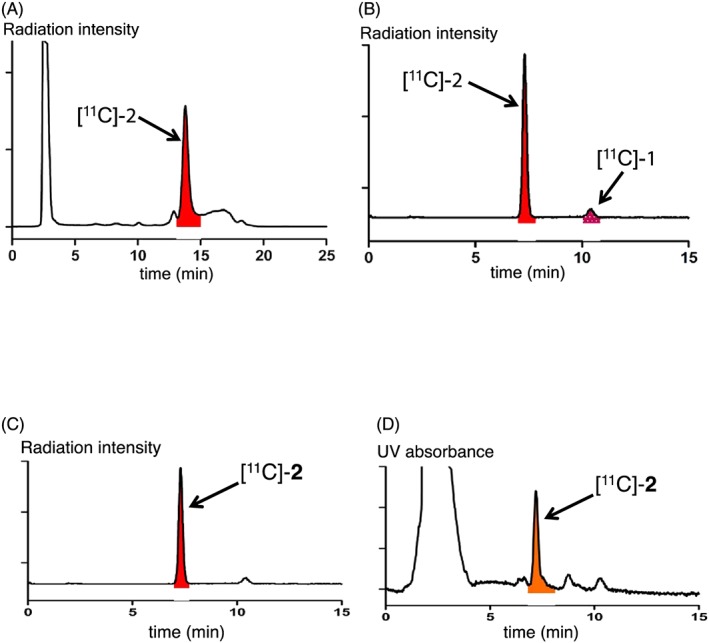
HPLC profiles of the reaction mixture and purity analysis during the synthesis of ^11^C‐labeled ubiquinol [^11^C]‐**2**. A, Semipreparative radio‐HPLC chromatogram; B, radio‐HPLC analysis of the sampling solution immediately after the HPLC purification of [^11^C]‐**2**; C, purity analysis of the injectable solution of [^11^C]‐**2** by radio‐HPLC; and D, purity analysis of the injectable solution of [^11^C]‐**2** by UV‐HPLC; the large peak around 0.5 to 4.5 min is mainly attributed to the use of ascorbic acid that is used as an additive

To synthesize [^11^C]‐**2**, [^11^C]‐**1** isolated by semipreparative HPLC was concentrated under reduced pressure and then reacted with Na_2_S_2_O_4_ at 75°C for 6 minutes. As shown in Figure 5A (semipreparative radio‐HPLC chromatogram of the reaction mixture), the desired peak of ^11^C‐labeled ubiquinol [^11^C]‐**2**, as a reduced product, was observed. However, we encountered difficulties in concentrating the solution of [^11^C]‐**2** after HPLC purification and preparing an injectable solution because [^11^C]‐**2** underwent decomposition immediately via radiolysis or oxidation. Ubiquinol is generally known as an essential radical acceptor and an antioxidant substrate. Therefore, it is easily oxidized to ubiquinone as a redox oxide. Probably, radicals, which are generated by radiolysis under PET chemistry conditions, cause the decomposition to produce ^11^C‐labeled ubiquinol [^11^C]‐**1**. In fact, during the HPLC analysis of [^11^C]‐**2**, the production of [^11^C]‐**1** was observed (Figure 5B).

In order to protect [^11^C]‐**2** from both radiolysis and oxidation, we employed ascorbic acid as a radical scavenger and an antioxidant.^28^ Ascorbic acid was added to all the used solutions, such as the HPLC eluent, any remaining solution inside the rotary evaporator, and injection solution, which were necessary for the purification of [^11^C]‐**2**. Fortunately, this tactic showed visible effects in many cases but did not always suppress the decomposition of [^11^C]‐**2**. This may be because [^11^C]‐**2** might probably undergo oxidation or change into a radical complex more easily, even in the presence of ascorbic acid. After radiopharmaceutical formulation using a solution (2.5 mL) of saline, propylene glycol, and Tween 80 (volume ratio, 100/10/1), including ascorbic acid (5 mg), the radioactivity and molar activity of [^11^C]‐**2** at the end of the synthesis were found to be 1.2 GBq in normal cases (obtained radioactivity range: 0.16‐1.4 GBq) and 48 to 76 GBq/μmol, respectively. The radiochemical purity and chemical purities of the obtained product were greater than 95% and around 77%, respectively, which were calculated by the ratios of the corresponding peak areas in analytical HPLC chromatogram. (Figure 5C and 5D). Synthesis was accomplished within 38 minutes, and the radiochemical yield based on [^11^C]CH_3_I was approximately 39%. The pH value was approximately 7. These characteristics of [^11^C]‐**2** make it suitable for animal PET studies. Currently, we are investigating the HPLC conditions to increase chemical purity of [^11^C]‐**2** for clinical PET studies in the future.

Solutions of [^11^C]‐**1** and [^11^C]‐**2** with a radioactivity of 75 MBq were intravenously administered via the tail veins of 8‐week‐old male Sprague‐Dawley rats. Whole‐body PET imaging was performed, and the accumulation levels of the PET probes were investigated. Interestingly, significant differences were observed between [^11^C]‐**1** and [^11^C]‐**2** in terms of their in vivo kinetics and biodistribution. These biological studies will be reported separately in a forthcoming paper.

## 3 CONCLUSIONS

We successfully synthesized ^11^C‐labeled ubiquinone [^11^C]‐**1** and ubiquinol [^11^C]‐**2** via Pd‐mediated rapid *C*‐[^11^C]methylation and a subsequent reduction reaction. The organoborane precursor **3** necessary for Pd‐mediated rapid *C*‐[^11^C]methylation was prepared by metathesis using commercially available natural ubiquinone. Thus, this procedure proves that unradiolabeled ubiquinone can be converted to ^11^C‐radiolabeled ubiquinone and ubiquinol. Biological studies using [^11^C]‐**1** and [^11^C]‐**2** are currently in progress. After synthesizing highly pure [^11^C]‐**2** (greater than 98%), we will move on to clinical PET studies as our ultimate goal.

## 4 EXPERIMENTAL SECTION

### 4.1 General methods

All reagents and solvents were used as received from their commercial sources without further purification. LC/TOF‐MS (Bruker Daltonik UHR‐TOF maXis 4G) was conducted to estimate the prenyl unit chain length of the products by the metathesis reaction of ubiquinone. Gel‐permeation chromatography was used to purify the desired pinacolboryl precursor **3** from crude products with different chain lengths of the prenyl unit. Pd‐mediated *C*‐[^11^C]methylation was conducted in a lead‐shielded hot cell operated by remote control. [^11^C]Carbon dioxide was produced by a ^14^N(p,α)^11^C nuclear reaction using a 12‐MeV cyclotron and then converted to [^11^C]CH_3_I by treatment with lithium aluminum hydride followed by hydriodic acid using an automated radiolabeling system, which involved heating the reaction mixture, dilution, HPLC injection, fractional collection, evaporation, and sterile filtration. The radioactivity of the compound was quantified with a dose calibrator. Semipreparative purification and purity analysis by HPLC were performed on a system equipped with pumps and an ultraviolet (UV) absorption detector, while the radioactivity of the effluent was determined using a radio analyzer. The radiochemical yield of the desired ^11^C‐labeled product was shown as the conversion yield based on the initial radioactivity [^11^C]CH_3_I of the ^11^C‐labeling reaction, which was calculated with a decay correction of ^11^C (half‐life: 20.4 min). The radiochemical and chemical purities of the ^11^C‐labeled product were calculated by the ratios of the corresponding peak areas in the analytical HPLC chromatogram.

### 4.2 Preparation of the pinacolboryl precursor

#### 4.2.1 Typical olefin metathesis of ubiquinone 1 and 2‐(pinacolboryl)propene to yield the desired pinacolboryl precursor 3, 39‐demethyl‐39‐(pinacolboryl)ubiquinone, (entry 3 in Table 1)

To a mixture of ubiquinone **1** (1.0 g, 1.2 mmol), a second‐generation Hoveyda‐Grubbs catalyst (22.5 mg, 36 μmol) in toluene (12 mL) and 2‐(pinacolboryl)propene (0.8 g, 4.8 mmol) were added. The reaction flask was evacuated and filled with N_2_ gas four times, after which the mixture was heated at 70°C for 2 hours. After cooling the reaction mixture to room temperature, a metal‐ion scavenger (QuadraSil AP) was added and the mixture was stirred for 1 hour. The suspended solution was filtered and washed with ethyl acetate. After removing the solvent under reduced pressure, a part of the residue was analyzed by LC/TOF‐MS (Figure 2). Most of the residue was purified by flash‐column chromatography on silica gel (hexane and a 30:1 solution of hexane and ethyl acetate) to yield a crude product. The crude product was further purified by gel‐permeation chromatography using tetrahydrofuran (THF) as the eluent and flash‐column chromatography on silica gel to afford the desired compound, 39‐demethyl‐39‐(pinacolboryl)ubiquinone **3**, as a colorless oil (up to 16 % yield, normal cases after the severe purification: 54 mg, 5% yield). Analytical HPLC; YMC‐Pack Pro C18 150 × 4.6 mm I.D., eluent: methanol/hexane = 9:1, flow rate: 1.0 mL/min, UV: 290 nm, column temperature: 30°C, retention times of **3**: 18.2 and 19.5 minutes for *trans* and *cis* isomers, respectively, at a ratio of 11:1 (Figure 3). ^1^H nuclear magnetic resonance (NMR) (CDCl_3_): *δ* 0.26 (s, 12H), 1.58–1.61 (m, 23H), 1.68 (s, 3H), 1.74 (s, 3H), 1.94–2.09 (m, 38H), 2.21 (td, *J* = 7.4, 7.4 Hz, 2H), 3.18 (d, *J* = 6.8 Hz, 2H), 3.98 (s, 3H), 3.99 (s, 1H), 4.94 (dd, *J* = 7.1, 7.1 Hz, 1H), 5.05–5.13 (m, 8H), 6.31 (td, *J* = 6.8, 1.4 Hz, 1H), ^13^C NMR (CDCl_3_): *δ* 11.9 (1CH_3_), 13.8 (1CH_3_), 14.1 (1CH_3_), 16.0 (6CH_3_), 16.3 (1CH_3_), 22.6 (1CH_2_), 24.77 (4CH_3_), 24.83 (1CH_3_), 25.3 (2CH_2_), 26.5 (2CH_2_), 26.7 (5CH_2_), 27.5 (2CH_2_), 31.6 (1CH_2_), 38.6 (2CH_2_), 39.7 (4CH_2_), 61.1 (2CH_3_), 83.0 (2C), 118.8 (1CH), 123.8 (1CH), 124.1 (1CH), 124.22 (4CH), 124.24 (1CH), 124.4 (1CH), 134.7 (1C), 134.90 (1C), 134.94 (4C), 134.98 (1C), 135.2 (1C), 137.6 (1C), 138.8 (1C), 141.7 (1C), 144.2 (1C), 144.3 (1C), 146.2 (1CH), 183.9 (1C), and 184.8 (1C). Signals corresponding to the carbon (39th position) attached to the boron atom were not observed.

### 4.3 Radiochemistry

#### 4.3.1 Synthesis of ^11^C‐labeled ubiquinone [^11^C]‐1 by Pd‐mediated rapid *C*‐[^11^C]methylation

[^11^C]CH_3_I (20‐40 GBq) with a He stream was trapped in a solution of the pinacolboryl precursor **3** (2.6 mg, 2.7 μmol), Pd_2_(dba)_3_ (1.4 mg, 1.5 μmol), and P(*o*‐tolyl)_3_ (1.9 mg, 6.2 μmol) in DMF (450 μL) at room temperature. To analyze the reactivity and suitability of the ^11^C‐labeling system, a solution of the pinacolboryl precursor **3** (5.8 mg, 6.0 μmol), Pd_2_(dba)_3_ (2.9 mg, 3.17 μmol), and P(*o*‐tolyl)_3_ (3.8 mg, 12.5 μmol) in DMF (450 μL) was used. The resulting mixture was heated at 65°C by hot air for 4 minutes and then diluted with DMF (0.7 mL). The mixture was transferred to a reservoir vessel using a rinse solution of DMF (0.2 mL). After passing through a cotton filter, the mixture was injected into a semipreparative HPLC system (mobile phase: ethanol/methanol/H_2_O = 55/40/5; column: COSMOSIL 5C18 PAQ 10 × 150 mm; flow rate: 5.0 mL/min from 0 to 0.5 min and 5.5 mL/min from 0.5 to 30 min; retention time of [^11^C]‐**1**: 17.5‐19.0 min; see Figure 4A). The fraction of interest was collected in a flask containing H_2_O (2.0 mL) and concentrated under reduced pressure. In accordance with the radiopharmaceutical formulation, the desired product [^11^C]‐**1** was dissolved in a mixed solution (1.6 or 2.2 mL) of saline, propylene glycol, and Tween 80 (volume ratio: 100/10/0.5) to be collected in a sterilized vial. The collection operation was repeated twice. The total synthesis time including ^11^C‐labeling reaction, HPLC purification, and radiopharmaceutical formulation was less than 36 minutes. The radioactivity of the formulated injection solution of [^11^C]‐**1** was 1.5 GBq in normal cases (obtained radioactivity range: 0.4‐3.5 GBq). The molar activity at the end of the synthesis was calculated to be 21 to 78 GBq/μmol. The radiochemical yield based on [^11^C]CH_3_I was up to 53%. Both the radiochemical purity and chemical purity of the product were greater than 99% (Figure 4B and 4B; mobile phase: ethanol/methanol = 15/85; column: COSMOSIL 5C18 PAQ 4.6 × 150 mm; flow rate: 1.0 mL/min; UV detection for Figure 4C: 290 nm). The pH value of the formulated injection solution was approximately 7.

#### 4.3.2 Synthesis of ^11^C‐labeled ubiquinol [^11^C]‐2

After heating the above‐described mixture for Pd‐mediated rapid *C*‐[^11^C]methylation, which consisted of [^11^C]CH_3_I and the pinacolboryl precursor **3**, for 4 minutes at 65°C, an aqueous solution (0.1 mL) of Na_2_S_2_O_4_ (5.0 mg, 21.5 μmol) was added as a reducing agent and the resulting mixture was further heated at 75°C by hot air for 6 minutes. The reaction mixture was diluted with DMF (0.7 mL) and then transferred to a reservoir vessel using a rinse solution of DMF (0.2 mL). After passing through a cotton filter, the mixture was injected into a semipreparative HPLC system (mobile phase: ethanol/methanol/H_2_O = 55/40/5 or ethanol/methanol/1% aqueous solution of ascorbic acid = 55/40/5; column: COSMOSIL 5C18 PAQ 10 × 150 mm; flow rate: 5.0 mL/min from 0 to 0.5 min and 5.5 mL/min from 0.5 to 30 min; UV detection: 290 nm; retention time of [^11^C]‐**2**: 13.5‐14.5 minutes; see Figure 5A). The fraction of interest was collected in a flask containing H_2_O (2.0 mL) and ascorbic acid (5.0 mg, 28.3 μmol) followed by concentration under reduced pressure to remove the solvent. In accordance with the radiopharmaceutical formulation, [^11^C]‐**2** was dissolved in a mixed solution (1.6 or 2.2 mL) of saline, propylene glycol, and Tween 80 (volume ratio: 100/10/0.5) to be collected in a sterilized vial. The collection operation was repeated twice. The final volume collected in the vial was 3.0 to 4.0 mL in normal cases. The total synthesis time, including the ^11^C‐labeling reaction, reduction reaction, HPLC purification, and radiopharmaceutical formulation, was less than 38 minutes. The radioactivity of the formulated injection solution of [^11^C]‐**2** was 1.2 GBq in normal cases (obtained radioactivity range: 0.16‐1.4 GBq), and the molar activity at the end of the synthesis was calculated to be 48 to 76 GBq/μmol. The radiochemical yield based on [^11^C]CH_3_I was approximately 39%. The radiochemical purity and chemical purity of ^11^C‐labeled ubiquinol [^11^C]‐2 were greater than 95% and around 77%, respectively (Figure 5C and 5D; mobile phase: ethanol/methanol = 15/85; column: COSMOSIL 5C18 PAQ 4.6 × 150 mm; flow rate: 1.0 mL/min; UV detection for Figure 5D: 290 nm). The pH value of the formulated injection solution was approximately 7.

HPLC analysis conditions for Figure 5B; mobile phase: ethanol/methanol = 15/85; column: COSMOSIL 5C18 PAQ 4.6 × 150 mm; flow rate: 1.0 mL/min.
